# A Single Amino Acid at Position 431 of the PB2 Protein Determines the Virulence of H1N1 Swine Influenza Viruses in Mice

**DOI:** 10.1128/JVI.01930-19

**Published:** 2020-03-31

**Authors:** Chengzhi Xu, Bangfeng Xu, Yunpu Wu, Shiman Yang, Yunhui Jia, Wenhua Liang, Dawei Yang, Likun He, Wenfei Zhu, Yan Chen, Huanliang Yang, Benliang Yu, Dayan Wang, Chuanling Qiao

**Affiliations:** a State Key Laboratory of Veterinary Biotechnology, Harbin Veterinary Research Institute, Chinese Academy of Agricultural Sciences, Harbin, People’s Republic of China; b Liaoning Provincial Agricultural Development Service Center, Shenyang, People’s Republic of China; c National Institute for Viral Disease Control and Prevention, Chinese Center for Disease Control and Prevention, Beijing, People’s Republic of China; Icahn School of Medicine at Mount Sinai

**Keywords:** genetic basis, influenza virus, virulence

## Abstract

Genetic reassortments occurred continuously among multiple subtypes or genotypes of influenza viruses prevalent in pigs. Of note, some reassortant viruses bearing the internal genes of the 2009 pandemic H1N1 (2009/H1N1) virus sporadically caused human infection, which highlights their potential threats to human public health. In this study, we performed phylogenetic analysis on swine influenza viruses (SIVs) circulating in Liaoning Province, China. A total of 22 viruses, including 18 H1N1 and 4 H1N2 viruses, were isolated from 5,750 nasal swabs collected from pigs in slaughterhouses from 2014 to 2016. H1N1 viruses formed four genotypes, which included Eurasian avian-like H1N1 (EA H1N1) and double/triple reassortant H1N1 derived from EA H1N1, 2009/H1N1, and triple reassortant H1N2 (TR H1N2) viruses. H1N1 SIVs with different genotypes and even those within the same genotypes represented different pathogenicities in mice. We further characterized two naturally isolated H1N1 SIVs that had similar viral genomes but differed substantially in their virulence in mice and found that a single amino acid at position 431 in the basic polymerase 2 (PB2) protein significantly affected the viral replication capacity and virulence of these two viruses. Taken together, our findings revealed the diverse genomic origins and virulence of the SIVs prevalent in Liaoning Province during 2014 to 2016, which highlights that continuous surveillance is essential to monitor the evolution of SIVs. We identified a naturally occurring amino acid mutation in the PB2 protein of H1N1 SIVs that impacts the viral replication and virulence in mice by altering the viral polymerase activity.

**IMPORTANCE** The frequent reassortment among different influenza viruses in pigs adds complexity to the epidemiology of swine influenza. The diverse viral virulence phenotypes underline the need to investigate the possible genetic determinants for evaluating the pandemic potential to human public health. Here, we found that multiple genotypes of influenza viruses cocirculate in the swine population in Liaoning Province, China. Furthermore, we pinpointed a single amino acid at position 431 in the PB2 protein which plays a critical role in the virulence of H1N1 viruses in mice and found that the alteration of viral polymerase activities is the cause of the different virulence. Our study further indicated that the virulence of influenza virus is a polygenic trait, and the newly identified virulence-related residue in the PB2 provides important information for broadening knowledge on the genetic basis of viral virulence of influenza viruses.

## Abstract (precis)

The frequent reassortment among different influenza viruses in pigs adds complexity to the epidemiology of swine influenza. The diverse viral virulence phenotypes underline the need to investigate the possible genetic determinants for evaluating the pandemic potential to human public health. Here, we found that multiple genotypes of influenza viruses cocirculate in the swine population in Liaoning Province, China. Furthermore, we pinpointed a single amino acid at position 431 in the PB2 protein which plays a critical role in the virulence of H1N1 viruses in mice and found that the alteration of viral polymerase activities is the cause of the different virulence. Our study further indicated that the virulence of influenza virus is a polygenic trait, and the newly identified virulence-related residue in the PB2 provides important information for broadening knowledge on the genetic basis of viral virulence of influenza viruses.

## INTRODUCTION

Swine influenza virus (SIV), first known as classical swine H1N1 (CS H1N1) virus, was introduced into pigs in 1918 in North America (1, 2). Eurasian avian-like H1N1 (EA H1N1) SIV was transmitted from poultry to pigs in 1979 in Europe (3). Triple reassortant H1N2 (TR H1N2) was isolated from pigs in 1999 in North America, which was derived from CS H1N1 and triple reassortant H3N2 viruses that have emerged among American pigs since 1998 (4). In 2009, further reassortment between EA H1N1 and TR H1N2 viruses occurred in pigs, which generated the novel variant virus (2009 pandemic H1N1 [2009/H1N1]) causing a human influenza pandemic worldwide (5, 6). After emergence of 2009/H1N1 virus in 2009, it quickly reintroduced into pigs (7). Rapidly, numerous outbreaks in swine were reported worldwide, including Europe and Asia (8–11). Pigs are considered a mixing vessel for the generation of such viruses, with genetic material originating from pigs, humans, and/or birds (12). Since 2009, genetic reassortment among the pandemic 2009/H1N1 virus, EA H1N1, and other swine H1N1, H1N2, and H3N2 viruses was detected repeatedly in pigs (13–20). Notably, sporadic human infections of endemic and novel reassortant SIVs (21–27) and the seropositivity of at-risk populations, such as swine workers (28–30), underscore the importance of influenza surveillance in pigs.

In China, an intensively active surveillance program for SIVs has been conducted since 2009. EA H1N1 viruses are the predominant subtype of influenza viruses in swine (31). A variety of reassortant viruses were also detected in pigs in southern China, which has been considered an epicenter of influenza viruses (14, 32). Few data are available about the prevalence of SIVs in Liaoning Province, which is in the northeast of China, despite it being the main swine-producing region in China (11, 33). Therefore, in this study, we conducted continuous surveillance for influenza viruses in pigs from 2014 to 2016 and analyzed the genetic and pathogenic characteristics of these isolates. Furthermore, we investigated the molecular determinants of two genetically similar viruses showing different virulence in mice and explored the possible underlying mechanism.

## RESULTS

### Multiple genotypes of H1N1 SIVs cocirculated in pigs.

From November 2014 to December 2016, a total of 5,750 nasal swabs were collected from pigs in slaughterhouses located in Liaoning Province, China. In total, 22 viruses, including 18 H1N1 and 4 H1N2, were isolated and identified. The eight gene segments of the isolates were sequenced.

**FIG 1:**
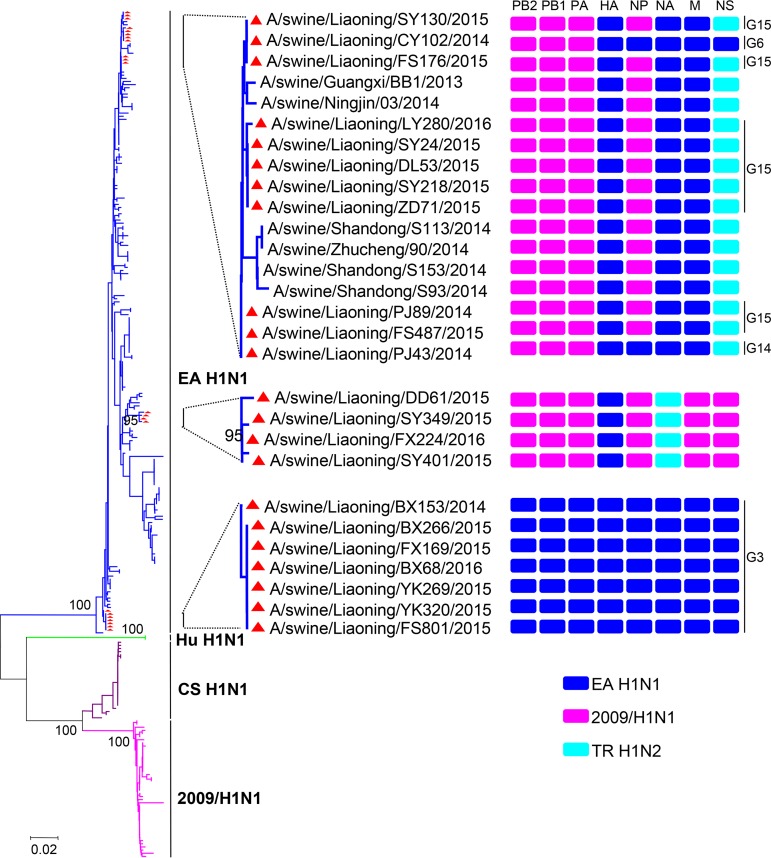
Genetic relationships among the HA genes and genotype analysis of H1N1 SIVs. The unrooted tree was constructed using the neighbor-joining method with the maximum composite likelihood model and MEGA7 (www.megasoftware.net) with 1,000 bootstrap replicates, based on nucleotide positions 33 to 1733. Scale bar indicates the number of nucleotide substitutions per site. The red triangles indicate viruses characterized in this study. EA, Eurasian avian-like; CS, classical swine; Hu, human; TR, triple reassortant. The colors of the bars represent the groups in the tree.

**FIG 2:**
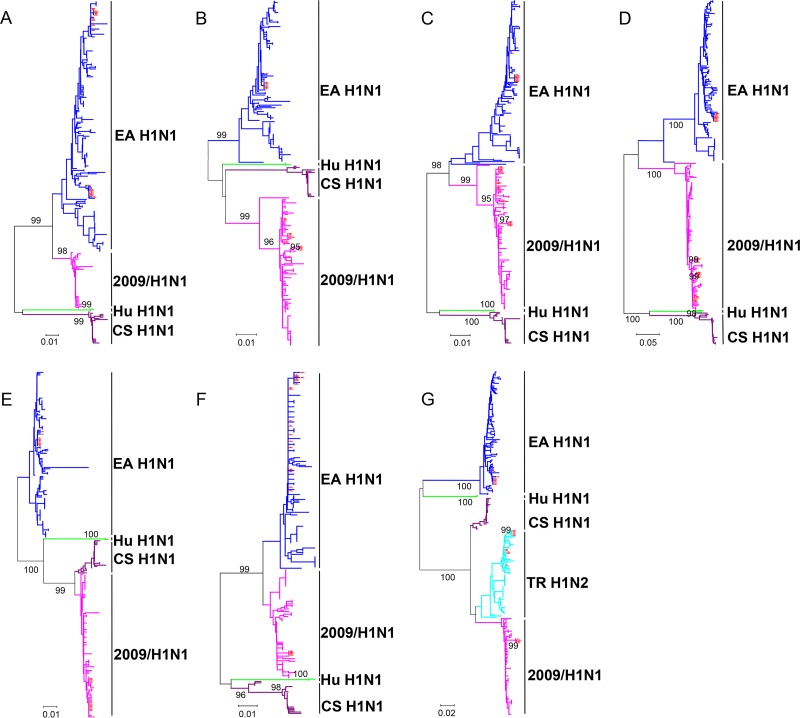
Phylogenetic analyses of the other seven genes of H1N1 SIVs. The unrooted trees were based on nucleotide positions 21 to 1430 for N1 NA (A), 28 to 2307 for PB2 (B), 25 to 2298 for PB1 (C), 25 to 2175 for PA (D), 46 to 1542 for NP (E), 26 to 1007 for M (F), and 27 to 864 for NS (G). Scale bar indicates the number of nucleotide substitutions per site. The red triangles indicate viruses characterized in this study. EA, Eurasian avian-like; CS, classical swine; Hu, human; TR, triple reassortant.

To characterize the gene segments of these viruses, we constructed unrooted phylogenetic trees using the gene sequences of 247 H1N1 reference strains previously isolated in mainland China, which were downloaded from the global initiative on sharing all influenza data (GISAID) (https://platform.gisaid.org/) until July 2019. Phylogenetic analysis of the hemagglutinin (HA) genes showed that all the analyzed viruses were classified into four lineages, including EA H1N1, human H1N1 (Hu H1N1), CS H1N1, and 2009/H1N1. The 22 viruses isolated in this study shared 95.2% to 100.0% sequence identity at the nucleotide level and fell into the EA H1N1 lineage (Fig. 1). Analysis of the N1 neuraminidase (NA) genes showed that all the H1N1 viruses isolated in this study belonged to the EA H1N1 lineage, with the nucleotide sequence identity of 95.2% to 100.0% (Fig. 2A). The N2 NA genes of the four H1N2 isolates shared the nucleotide sequence identity of 99.8% to 99.9% and were most closely related to a H1N2 virus (A/swine/Guangxi/325/2012) previously isolated in China, which clustered into the North American TR H1N2 lineage (15). Twenty-two viruses were clustered into two distinct lineages based on their basic polymerase 2 (PB2) genes, in which the 2009/H1N1 lineage included 11 H1N1 and 4 H1N2 viruses and the EA H1N1 lineage included 7 H1N1 viruses (Fig. 2B). The genetic trees constructed for the basic polymerase 1 (PB1) and acidic polymerase (PA) genes were similar to that for the PB2 gene, which showed that 11 H1N1 and 4 H1N2 viruses clustered into the 2009/H1N1 lineage and 7 H1N1 viruses clustered into the EA H1N1 lineage (Fig. 2C and D). In the phylogenetic tree for the nucleoprotein (NP) gene, 9 H1N1 and 4 H1N2 viruses clustered into the 2009/H1N1 lineage, and another 9 H1N1 viruses belonged to the EA H1N1 lineage (Fig. 2E). In the matrix (M) gene tree, 18 H1N1 viruses all belonged to the EA lineage, with a nucleotide identity ranging from 94.2% to 100%. Furthermore, 4 H1N2 viruses were clustered into the 2009/H1N1 sublineage (Fig. 2F). Phylogenetic analysis of the nonstructural protein (NS) genes showed that the viruses isolated in this study were classified into three lineages, in which the EA H1N1 lineage included 8 H1N1 viruses, the TR H1N2 lineage included 10 H1N1 viruses, and the 2009/H1N1 lineage included 4 H1N2 viruses (Fig. 2G).

**FIG 3:**
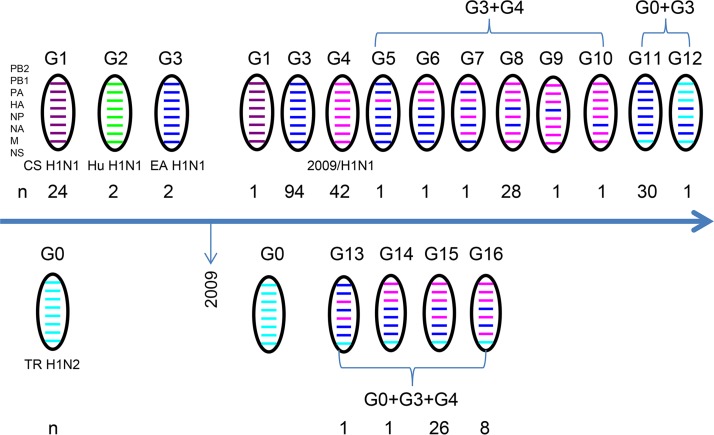
Genotype evolution of the H1N1 SIVs isolated in mainland China. The whole-genome sequences analyzed here include 18 H1N1 viruses isolated in this study and 247 H1N1 reference strains downloaded from GISAID (https://platform.gisaid.org/) until July 2019. *n*, number of the viruses belonging to the indicated genotype.

To further provide a systematic evolutionary analysis of the H1N1 SIVs isolated in mainland China, we analyzed the genomic sequences of all the 247 H1N1 reference strains and 18 H1N1 viruses isolated in this study (Fig. 3). To date, a total of 16 genotypes of H1N1 SIVs have been reported. Prior to 2009, CS H1N1 viruses were predominantly isolated from pigs, and Hu H1N1 and EA H1N1 viruses were only sporadically isolated. After 2009, EA H1N1 and 2009/H1N1 viruses were repeatedly isolated from pigs. Apart from H1N1, the TR H1N2 virus was another important subtype of influenza virus that consistently circulated in the swine population. Since the emergence of the 2009/H1N1 virus in pigs, pure 2009/H1N1 viruses were only isolated from 2009 to 2011 but continuously provided one or more internal genes to other endemic SIVs. Genetic reassortment occurred frequently between different influenza viruses. Here, six and two types of double reassortant H1N1 viruses were detected between EA H1N1 and 2009/H1N1 and between EA H1N1 and TR H1N2 viruses, respectively, during 2010 and 2014. Furthermore, four types of triple reassortant H1N1 viruses among EA H1N1, 2009/H1N1, and TR H1N2 were isolated from pigs during 2013 and 2016 (Fig. 3). Also, as shown in Fig. 1, 18 H1N1 viruses isolated in this study were classified into four genotypes, including G3, G6, G14, and G15, which indicates that multiple genotypes of H1N1 SIVs cocirculated in pigs in Liaoning Province.

### Diverse pathogenicities of the isolates in mice.

**FIG 4:**
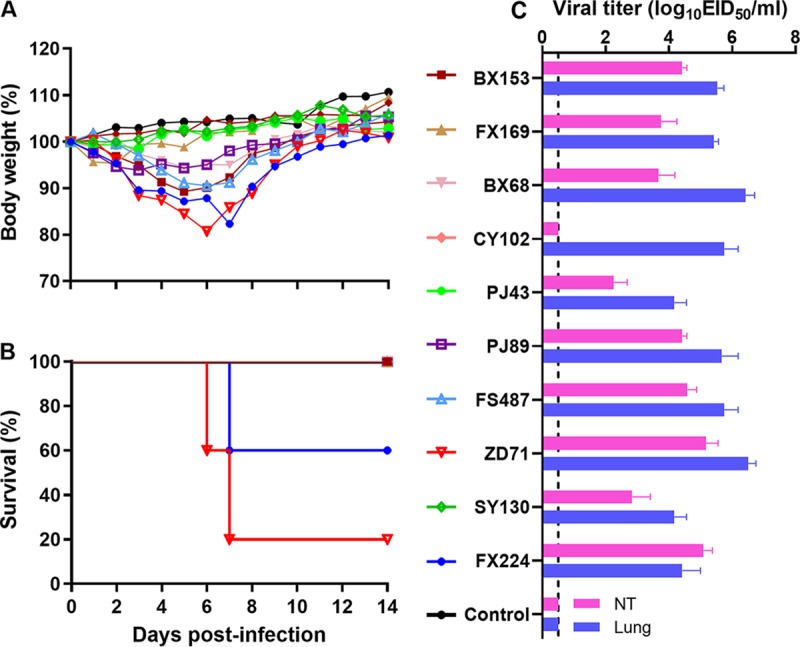
Pathogenicity of the viruses in mice. (A) Weight changes, (B) survival curves, and (C) virus titration in the NTs and lungs of the mice after viral infection. Groups of mice were i.n. infected with 10^5^ EID_50_ of the indicated viruses in a volume of 50 μl, and mice in the control group were i.n. inoculated with 50 μl of PBS. The body weights of all the mice were monitored daily for 2 weeks. Values indicate the mean weight changes of all the mice in each group after infection. At 3 dpi, the lungs and NTs were collected and titrated in eggs. Values indicate the mean viral titers in the lungs or NTs of three mice and are expressed as the mean ± SD. Samples for which the virus was not detected were assigned a numeric value of 0.5 for calculation purposes. The dashed horizontal line indicates the limit of detection.

To evaluate the virulence of the 10 representative isolates, groups of mice were intranasally (i.n.) inoculated with 10^5^ 50% egg infective dose (EID_50_) of the indicated viruses. Compared with the control group, all the infected mice, except those inoculated with A/swine/Liaoning/CY102/2014 (H1N1) (CY102) and A/swine/Liaoning/SY130/2015(H1N1) (SY130) viruses, showed various degrees of weight loss within 1 week after infection, with the average ratio of decline ranging from 1.6% to 19.3%, respectively (Fig. 4A). In particular, four of the mice in the group infected with the A/swine/Liaoning/ZD71/2015 (H1N1) (ZD71) virus and two mice infected with the A/swine/Liaoning/FX224/2016 (H1N2) (FX224) virus showed weight loss with the ratio exceeding 25% and were, therefore, considered dead during the observation period after infection (Fig. 4B). At 3 days postinfection (dpi), no virus was detected in the brain, spleen, or kidneys of the mice infected with all the viruses (data not shown). Viruses were simultaneously detected in the nasal turbinates (NTs) and lungs of mice infected with nine viruses, with the average viral titers ranging from 2.25 log_10_ EID_50_/ml to 5.17 log_10_ EID_50_/ml in the NTs and from 4.17 log_10_ EID_50_/ml to 6.5 log_10_ EID_50_/ml in the lungs, respectively. Interestingly, CY102 was only detected in the lungs with a titer of 5.75 log_10_ EID_50_/ml, but not in the NT at 3 dpi (Fig. 4C). These results indicate that variations in virulence and replication capability exist among these H1N1 SIVs in mice. At the end of the experiment, all infected mice seroconverted, with the hemagglutination inhibition (HI) antibody titers ranging from 80 to 640, which indicated that these viruses could directly infect mice without adaptation.

### Rescued SY130 and ZD71 viruses maintained the properties of their parental wild-type viruses.

**FIG 5:**
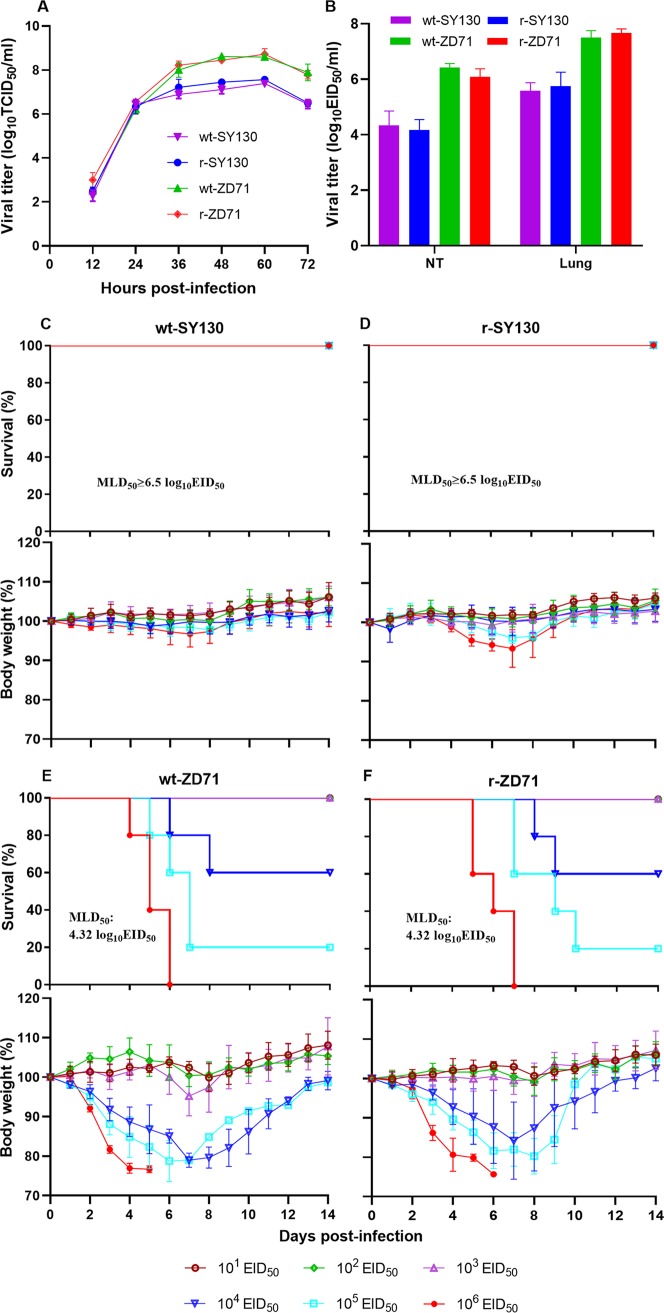
Establishment of RGSs for the SY130 and ZD71 viruses. (A) Growth kinetics in MDCK cells, (B) replication capacity in mice, and (C to F) MLD_50_ determination of the rescued and parental viruses. MDCK cells (MOI, 0.001) were inoculated with wt-SY130, r-SY130, wt-ZD71, and r-ZD71. Supernatants were collected at 12-h intervals until 72 hpi and titrated in MDCK cells by TCID_50_. In each panel, the average of three replicates is shown, with error bars indicating the SD. Groups of three mice were i.n. inoculated with 10^6^ EID_50_ (in a 50 μl volume) of the indicated viruses. At 3 dpi, the NTs and lungs were collected and titrated in eggs. Groups of five mice were inoculated with doses of 10^1^ to 10^6^ EID_50_ of virus in a 50-μl volume, and MLD_50_ values were calculated using the method of Reed and Muench.

As described above, the viruses SY130 and ZD71 shared similar genetic backgrounds and belonged to the same genotype but displayed substantially different levels of virulence in mice. SY130 only induced slight weight loss within 2 weeks after infection, whereas ZD71 showed higher virulence in mice, causing death in four out of five mice, as evaluated by 25% weight loss. To explore the molecular basis for these differences in virulence in mice, eight plasmid reverse genetic systems (RGSs) were established for the SY130 and ZD71 viruses, and both recombinant viruses (designated r-SY130 and r-ZD71) were rescued and confirmed by sequence analysis. Growth in MDCK cells and replication capacity in mice showed that the rescued viruses shared the same replication characteristics as their parental wild-type (wt) viruses (Fig. 5A and B). In addition, the 50% mouse lethal dose (MLD_50_) was determined for both the rescued and parental wt viruses. The MLD_50_ values for the r-SY130 and wt-SY130 viruses were both above 6.5 log_10_ EID_50_, while the MLD_50_ values of for the r-ZD71 and wt-ZD71 viruses were 4.32 log_10_ EID_50_ (Fig. 5C to F). These results demonstrated that both of the rescued viruses maintained the properties of their parental wt viruses in terms of viral replication and virulence in mice.

### The PB2 gene plays a major role in the difference in the pathogenicity between SY130 and ZD71 viruses.

**FIG 6:**
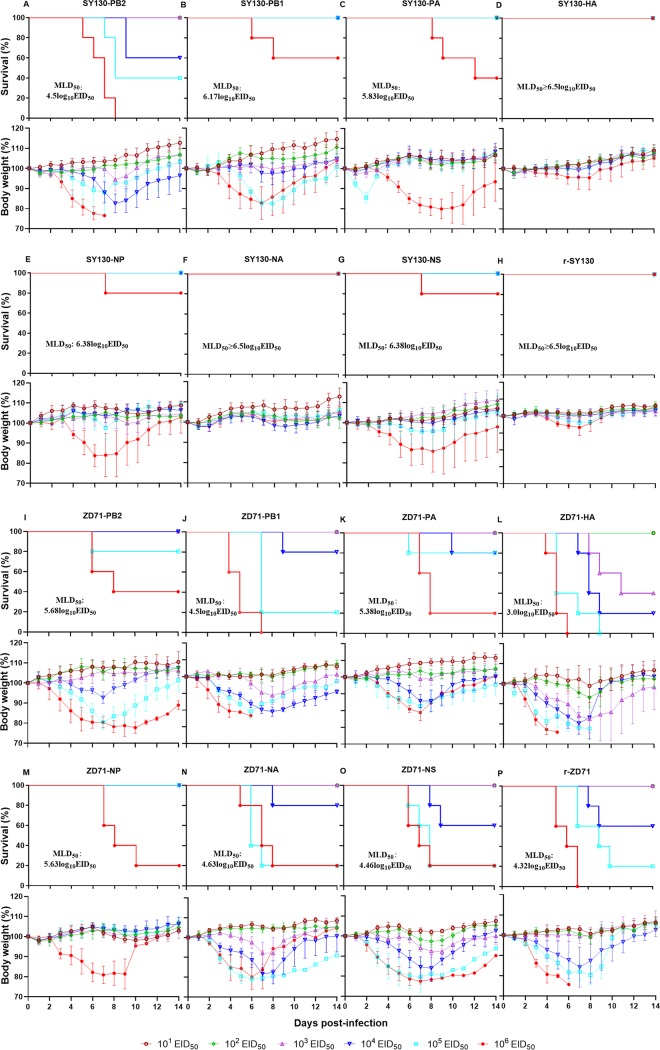
The reassortant viruses generated by single-gene substitutions and their virulence in mice. MLD_50_ determination of the reassortant viruses generated with SY130 (A to H) and ZD71 (I to P) as background. Groups of five mice were i.n. inoculated with doses of 10^1^ to 10^6^ EID_50_ of virus in a 50-μl volume, and MLD_50_ values were calculated using the method of Reed and Muench.

Based on the respective genetic alignments of SY130 and ZD71, we found that these two viruses shared the same M protein sequence but differed in their PB2, PB1, PA, HA, NP, NA, and NS proteins at the amino acid level. As shown in Table 1, we listed all the amino acids which were different between the two viruses. To identify which of these gene segments were responsible for the different virulence in mice, a total of 14 reassortant viruses were generated by exchanging a single gene between the r-SY130 and r-ZD71 viruses as described previously (34), and their MLD_50_ values were determined by measuring the weight loss of mice (Fig. 6A to G, and Fig. 6I to O). Compared with the parental r-SY130 virus, a single HA or NA gene substitution introduced into the SY130 virus did not affect virulence (Fig. 6D and F), whereas substitution of the NP, NS, or PB1 gene could increase the virulence to a certain extent (Fig. 6E, G, and B). The SY130-based reassortants carrying the PA and PB2 genes of ZD71 virus displayed virulence enhancement, with MLD_50_ values of 5.83 log_10_ EID_50_ and 4.5 log_10_ EID_50_, respectively (Fig. 6C and A). The ZD71-based reassortant viruses carrying a single PB1, NA, and NS gene segment from the SY130 virus showed similar MLD_50_ values to that of the parental r-ZD71 virus (Fig. 6J, N and O), whereas the reassortants carrying a single PB2, PA, and NP gene segment showed attenuated virulence in mice, with their MLD_50_ values increasing from 4.32 log_10_ EID_50_ to 5.68, 5.38, and 5.63 log_10_ EID_50_, respectively (Fig. 6I, K and M). Interestingly, we found that the HA gene of SY130 increased the virulence of the reassortant ZD71-HA virus to a certain extent, and its MLD_50_ value was 3.0 log_10_ EID_50_ (Fig. 6L). These results indicate that multiple genes determine the different viral pathogenicities of the ZD71 and SY130 viruses in mice, but the PB2 gene plays the most important role in the difference of viral pathogenicity between these two H1N1 viruses.

**TABLE 1:** Amino acid differences between the SY130 and ZD71 viruses^*a*^

Gene	Amino acid position	Amino acid carried by the indicated strain	
		SY130	ZD71
PB2	172	V	M
	431	T	M
	448	N	Y
	453	S	P
PB1	14	V	A
	216	G	D
PB1-F2	47	N	S
	83	F	S
PA	100	V	I
	651	S	A
PA-X	100	V	I
HA^*b*^	4	A	V
	138	S	A
	144	A	T
	225	E	G
NP	2	V	A
	384	R	K
NA	79	A	V
NS1	36	L	I
	56	T	A
	197	S	N
NS2	40	V	I
	72	K	R

a No amino acid differences were observed between the M genes of these two viruses.

b The amino acid positions of HA are H3 numbering.

### Identification of amino acid residues involved in the virulence difference between the two viruses.

**FIG 7:**
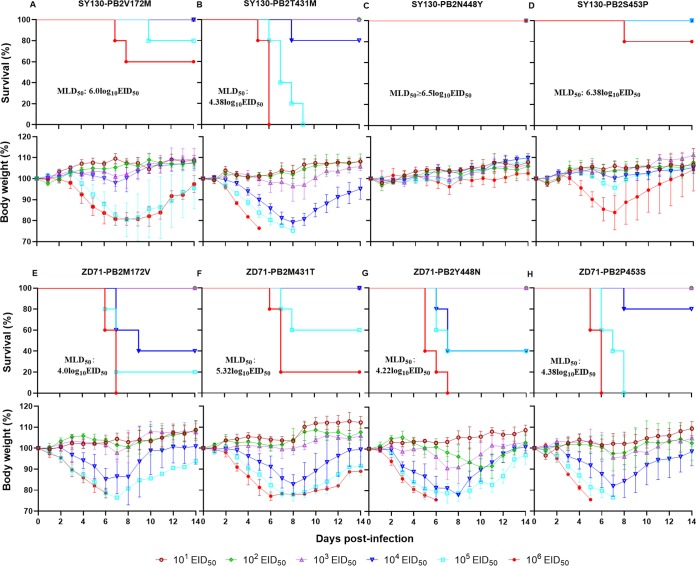
The reassortant viruses (PB2 mutant) generated by site-directed mutagenesis and their virulence in mice. MLD_50_ determination of the reassortant viruses generated with SY130 (A–D) and ZD71 (E–H) as background. Groups of five mice were i.n. inoculated with doses of 10^1^ to 10^6^ EID_50_ of virus in a 50-μl volume, and MLD_50_ values were calculated using the method of Reed and Muench.

According to the results obtained with the reassortant viruses generated by single-gene substitution, we found that the PB2 protein was a key viral protein determining the differences in virulence between the SY130 and ZD71 viruses in mice. As shown in Table 1, the PB2 proteins of the SY130 and ZD71 viruses differed at four amino acids at positions 172, 431, 448, and 453. Next, to determine which amino acid residue(s) contributed to the different virulence of SY130 and ZD71 viruses, we generated four mutant reassortant viruses by introducing the amino acid changes V172M, T431M, N448Y, and S453P individually into the r-SY130 virus and generated another four mutants by introducing the reverse mutations into the r-ZD71 virus. Then, we determined the MLD_50_ values of all the mutants and compared them with the parental r-SY130 and r-ZD71 viruses. The mutant SY130-PB2V172M, SY130-PB2N448Y, and SY130-PB2S453P viruses still showed low virulence, but the SY130-PB2T431M virus acquired high virulence in mice with the MLD_50_ value increasing to 4.38 log_10_ EID_50_ (Fig. 7A to D). Conversely, the mutant ZD71-PB2M431T became less virulent in mice (MLD_50_ of 4.32 log_10_ EID_50_ versus 5.32 log_10_ EID_50_) (Fig. 7F). These results demonstrate that the amino acid substitution at position 431 in the PB2 protein significantly affects the virulence of these two H1N1 viruses in mice.

### The amino acid at site 431 in the PB2 protein impacts viral replication *in vitro* and *in vivo*.

**FIG 8:**
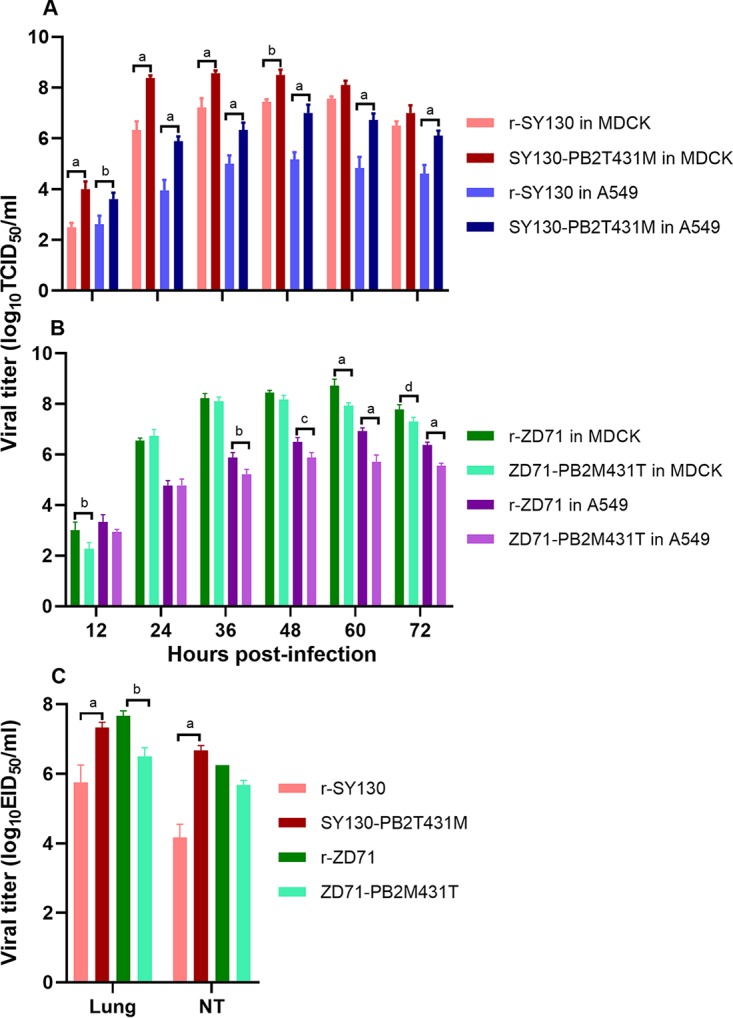
Effects of amino acid substitutions at residue 431 in PB2 on the viral replication capacity of mutant virus *in vitro* (A and B) and *in vivo* (C). MDCK cells (MOI, 0.001) or A549 cells (MOI, 0.1) were inoculated with r-SY130, SY130-PB2T431M, r-ZD71, and ZD71-PB2M431T. Supernatants were collected at 12-h intervals until 72 hpi and titrated in MDCK cells by TCID_50_. In each panel, the average of three replicates is shown, with error bars indicating the SD. Groups of three mice were i.n. inoculated with 10^6^ EID_50_ (in a 50-μl volume) of r-SY130, SY130-PB2T431M, r-ZD71, and ZD71-PB2M431T virus. At 3 dpi, the NTs and lungs were collected and titrated in eggs. Data shown are the mean viral titers ± SD. Statistical analysis was performed using two-way ANOVA, followed by Tukey’s multiple-comparison test. (a) *P* < 0.0001, (b) *P* < 0.001, (c) *P* < 0.01, and (d) *P* < 0.05 indicate titers compared with the corresponding viral replication titer of the parental and mutant viruses at the same time points or in the same organs of mice.

To determine whether the single amino acid at site 431 in the PB2 protein influences virus growth dynamics in MDCK or A549 cells, we compared the growth curves of SY130-PB2T431M and ZD71-PB2M431T with those of the respective parental r-SY130 and r-ZD71 viruses, respectively. When the T431M substitution was introduced into the r-SY130 virus, SY130-PB2T431M displayed significantly increased replication compared with the parental r-SY130 virus in MDCK cells during 12 and 48 hours postinfection (hpi), as well as in A549 cells at all the time points (*P* < 0.0001) (Fig. 8A). In contrast, the M431T substitution decreased replication of the ZD71-PB2M431T virus in MDCK cells, as well as in A549 cells, and the effects on the viral replication titer were mainly evident in the late stage of infection (*P* < 0.0001) (Fig. 8B). We further compared the replication capacities of SY130-PB2T431M and ZD71-PB2M431T in mice with those of the parental r-SY130 and r-ZD71 viruses, respectively. We found that the SY130-PB2T431M virus could be detected at a significantly higher titer than the parental r-SY130 virus, with the titer increasing from 5.75 to 7.33 log_10_ EID_50_/ml in the lungs and from 4.17 to 6.67 log_10_ EID_50_/ml in the NT (Fig. 8C). The titers of ZD71-PB2M431T in the lung were also significantly decreased compared with the parental r-ZD71 virus, with the titer decreasing from 7.67 to 6.5 log_10_ EID_50_/ml. The viral replication of ZD71-PB2M431T was also reduced compared with the wt virus in the NT, to some extent (Fig. 8C). These results further indicate that a single amino acid substitution at site 431 in the PB2 protein significantly affects the viral replication capacities of these viruses *in vitro* and *in vivo*.

### The amino acid residue at position 431 in the PB2 protein alters the viral polymerase activity.

**FIG 9:**
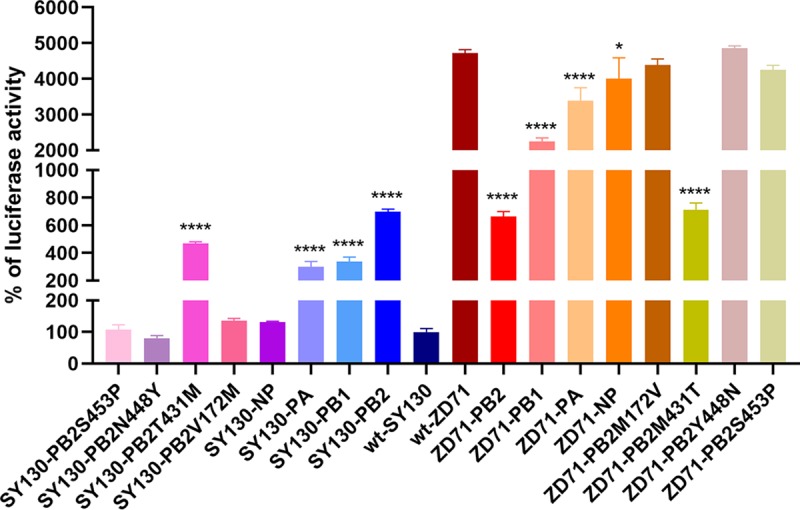
Viral polymerase activity of RNP complexes. HEK293T cells were transfected with the indicated PB2, PB1, PA, and NP genes in pcDNA3.1(+) together with the luciferase reporter plasmid pPolI-Luc. Cells were also cotransfected with a *Renilla* luciferase expression plasmid (pRL-TK) to control for transfection efficiency. As a control, samples lacking pcDNA3.1-NP were included. Twenty-four hours posttransfection, firefly and *Renilla* luciferase levels were measured, and *Renilla* expression was used to normalize the data. The relative activity of wt-SY130 RNP was set as 100%, and the relative activities of other reassortant RNPs were presented relative to that. Results shown are the averages with SD from three independent experiments. ****, *P* < 0.0001 and *, *P* < 0.05 indicate values compared with the corresponding values of polymerase activity of RNP carrying the parental and mutant PB2 protein.

To investigate how the PB2 gene affects the viral virulence of SY130 and ZD71 viruses, we applied a minigenome reporter assay to assess the impact of the amino acid at position 431 in the PB2 protein on the polymerase activity in HEK293T cells. We first compared the polymerase activities of the wt RNP of the SY130 and ZD71 viruses and found that the relative activity of ZD71 was more than 47-fold higher than that of SY130 (Fig. 9). Then, we tested the polymerase activity of the reconstituted RNP containing the single-gene substitution or mutant PB2 and compared it with that of the parental RNP of the SY130 and ZD71 viruses. As shown in Fig. 9, the PB2, PB1, PA, and NP genes of the ZD71 virus could increase the relative activities of the respective RNP of SY130-PB2, SY130-PB1, SY130-PA, and SY130-NP by approximately 7.0-, 3.4-, 3.0-, and 1.3-fold, which suggests that the PB2 gene had the most significant effect on viral polymerase activity. We further tested the relative activities of the mutant RNP by introducing the individual mutation into the PB2 gene of SY130 at positions 172, 431, 448, and 453, respectively. Compared with the wt-SY130, no significant change was observed for SY130-PB2V172M, SY130-PB2N448Y, and SY130-PB2S453P. However, PB2-431M in RNP significantly increased the polymerase activity of SY130-PB2T431M. Conversely, the PB2 gene of the SY130 virus and the PB2 431T mutation were associated with a significant decrease in polymerase activity of the ZD71-PB2 and ZD71-PB2M431T viruses in HEK293T cells (Fig. 9). These results indicate that the amino acid at position 431, but not those at positions 172, 448, and 453 in the PB2 protein, contributes to the difference in the polymerase activities of the SY130 and ZD71 viruses, which further suggests that the different virulence and replication capacity of these two viruses are due to their different viral polymerase activities.

### Amino acid analysis of site 431 in the PB2 protein of influenza A viruses.

To investigate the prevalence of 431M or 431T of the PB2 protein among influenza viruses, we downloaded the full-length and partial PB2 sequences from the NCBI Influenza Virus Resource database (https://www.ncbi.nlm.nih.gov/genomes/FLU/Database/nph-select.cgi?go=database) until 8 September 2019 and performed a comprehensive sequence analysis with regard to position 431 of the PB2 protein. As shown in Table 2, PB2-431M was highly conserved among all the available sequences of H1N1 viruses isolated from swine, avian, and human, and the percentages were 97.3%, 96.3%, and 98.5%, respectively. The amino acid variant 431T was exceedingly rare among the H1N1 isolates, with only one avian H1N1 virus carrying PB2-431T (Table 2). We further analyzed the percentages of PB2-431M and PB2-431T among all the available sequences of the other subtype influenza viruses, including H1N2, H3N2, H5N1, H7N9, and H9N2. The results also revealed high conservation of PB2-431M among these viruses. In particular, all the H1N2, H3N2, H5N1, and H7N9 viruses possessed PB2-431M, and only one avian H9N2 virus carried PB2-431T (Table 2).

**TABLE 2:** Amino acid frequencies at position 431 in the PB2 protein of different subtype influenza viruses^*a*^

Virus subtype (*n*)	Host (*n*)	Frequency (%) of amino acid at PB2 residue 431 (*n*)						
		M	X	-	T	L	K	V
H1N1 (17,013)	Swine (2,560)	97.3 (2,491)	0.4 (10)	2.3 (59)	/	/	/	/
	Avian (629)	96.3 (606)	/	3.5 (22)	0.2 (1)	/	/	/
	Human (13,824)	98.5 (13,616)	0.21 (29)	1.28 (177)	/	0.03 (2)	/	/
H1N2 (1,946)		95.7 (1,863)	/	4.3 (83)	/	/	/	/
H3N2 (21,830)		99.3 (21,678)	0.1 (12)	0.6 (140)	/	/	/	/
H5N1 (3,313)		96.8 (3,206)	0.1 (4)	3.1 (103)	/	/	/	/
H7N9 (855)		99.8 (853)	/	0.23 (2)	/	/	/	/
H9N2 (2,721)		93.3 (2,538)	0.3 (9)	6.3 (171)	0.04 (1)	/	0.04 (1)	0.04 (1)

a The amino acid frequencies were determined for unique PB2 full-length sequences and partial sequences collected using the indicated criteria from the NCBI Influenza Virus Resource database (https://www.ncbi.nlm.nih.gov/genomes/FLU/Database/nph-select.cgi?go=database). n, number of sequences analyzed; X, amino acid deletion; /, not found; -, not determined.

### M431T substitution in the PB2 protein attenuated the other H1N1 viruses in mice.

**FIG 10:**
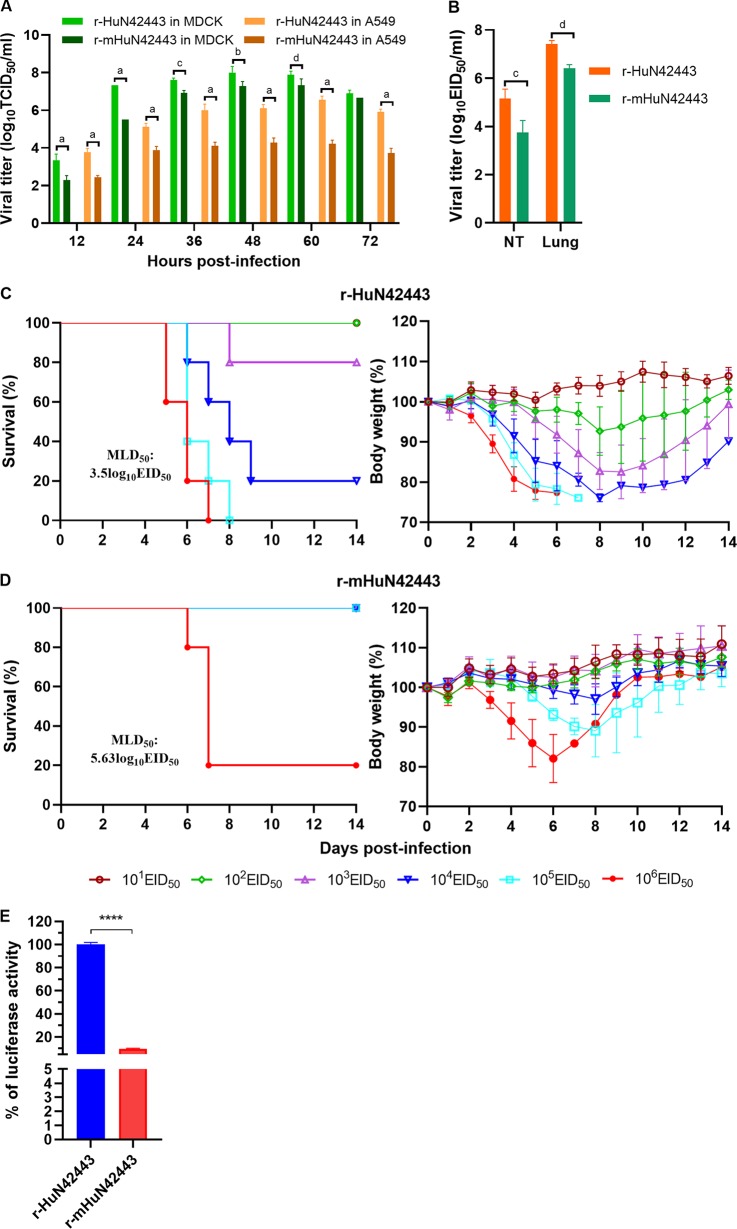
M431T substitution in the PB2 protein attenuated the virulence of HuN42443 virus in mice. Viral replication of the parental r-HuN42443 and mutant r-mHuN42443 virus in MDCK and A549 cells (A), as well as in mice (B); MLD_50_ determination of the parental r-HuN42443 and the mutant r-mHuN42443 virus (C, D); M431T substitution downregulated the polymerase activity of RNP of r-mHuN42443 (E). (a) *P* < 0.0001, (b) *P* < 0.001, (c) *P* < 0.01, and (d) *P* < 0.05 indicate titers compared with the corresponding viral titer of the parental and the mutant viruses at the same time point or in the same organs of mice. ****, *P* < 0.0001 indicates values compared with the corresponding values of polymerase activity of RNP carrying the parental and mutant PB2 protein.

To assess whether one particular residue was responsible for the attenuation of virulence in mice, we introduced the same amino acid change into a reassortant H1N1 virus (A/Hunan/42443/2015 [HuN42443]) previously isolated from human, which showed high virulence in C57BL/6J mice (26). In this study, we simultaneously rescued the parental HuN42443 (r-HuN42443) and mutant HuN42443 bearing the M431T substitution (r-mHuN42443) by using RGS and tested their replication capacities and pathogenicities in BALB/c mice. We found that the r-mHuN42443 virus displayed decreased replication both in MDCK and A549 cells, as well as *in vivo*, compared with the parental r-HuN42443 virus (Fig. 10A and B). Moreover, r-mHuN42443 also showed lower virulence in BALB/c mice than the parental r-HuN42443 virus, with the MLD_50_ value increasing from 3.5 to 5.63 log_10_EID_50_ (Fig. 10C and D). Polymerase activity was significantly decreased when the PB2 protein harboring M431T was introduced into the parental RNP complex (Fig. 10E). These results confirm that PB2 M431T is a major factor that affects the virulence of H1N1 SIV in mice.

## DISCUSSION

Genetic reassortment is considered the main mechanism for the emergence of novel influenza viruses to escape the preexisting immunity of the host. Previous studies reported that the pandemic 2009/H1N1 and EA H1N1 viruses have been isolated in pigs in Liaoning Province (31, 33, 35). In the present study, we examined the genetic composition of influenza viruses isolated during the active surveillance of pigs from 2014 to 2016 in this region. Our data indicated that four different genotypes of H1N1 SIVs coexisted in pigs, including the pure EA H1N1 (G3), double reassortant (G6), and triple reassortant H1N1 (G14 and G15). In addition, the TR H1N2 viruses were also isolated, which were genetically similar to the previously reported isolates (15). Among the reassortant H1N1 viruses isolated in our study, G6-like and G14-like viruses were only sporadically detected in 2014, while G15-like reassortants were repeatedly isolated between 2014 and 2016 in Liaoning Province. Recent surveillance results from other provinces in China confirmed the multiple genomic characteristics of SIVs (19, 20). Especially, G15-like viruses were continuously isolated, which suggested that these kinds of reassortant viruses have become established in the swine population. Of note, the TR H1N1 viruses (HuN42443) bearing G15-like genetic compositions have been detected in human (26), as well as in canine (36), in China. Further studies demonstrated that the human G15-like reassortant H1N1 virus showed higher virulence than human EA H1N1 virus in the mouse model, which highlights the potential risk of SIV to public health.

The pathogenicity of influenza virus in mammals is complex and determined by multiple viral genes (37–43). In this study, we simultaneously tested the pathogenicities of EA H1N1 and the reassortant viruses carrying the internal genes of the 2009/H1N1 virus in mice and found that all the viruses could replicate efficiently *in vivo* and caused certain weight losses, and some viruses led to severe disease or even the death of mice. Of interest, we found that a pair of viruses, namely, SY130 and ZD71, shared the highest sequence identity, ranging from 98.2% to 100%, for the eight gene segments but exhibited significantly different pathogenicity in mice. SY130 did not cause the apparent weight loss in mice when infected with 10^5^ EID_50_, whereas, the ZD71 virus caused significant weight loss in mice, which was clinically considered the cause of death. An alignment of the genomes of these two viruses showed that they differed by 23 amino acids spanning in the 7 gene fragments, with the exception of the M gene. This observation raised the possibility that as yet unknown molecular determinants may affect the virulence of SIVs in mice.

As an important component of the viral polymerase complex, the PB2 protein is a viral polymerase with 759-amino acid residues composed of an N-terminal domain, a middle domain, a cap-binding domain, a 627 domain, and an NLS domain (44, 45). Several residues in the PB2 protein have been previously identified as virulence markers as well as factors contributing to interspecies transmission of influenza A viruses in mammals (39, 46–49). In particular, some mutations, such as E627K, D701N, E158R, A271T, Q591R, H357N, T558I, K339T, A588T, I147T, and I292V (46, 47, 50–56), in the PB2 protein are essential to determine the pathogenesis of influenza viruses in mammals. In this study, we first demonstrated that the PB2 protein had a major effect on the viral pathogenicity of two H1N1 SIVs, with the other genes playing only minor roles in virulence. Then, we further identified the T431M amino acid substitution in the PB2 protein responsible for virulence difference in mice between SY130 and ZD71 viruses. We also evaluated the viral replication capacities *in vitro* and *in vivo* and their polymerase activities in mammalian cells. The results showed that the amino acid at site 431 in the PB2 protein significantly influences viral virulence and replication capacity *in vitro* and *in vivo* by affecting the polymerase activity of RNP. Our results support that amino acids in the cap-binding domain of PB2 specifically contributed to the replication process of avian influenza viruses in human cells and virulence in mice (57, 58). Finally, we verified our findings by introducing the M431T substitution into the PB2 protein of a human H1N1 virus (HuN42443), which shared the same genomic characteristics with the ZD71 virus. Similar results were observed in our study when evaluated by replication capacity, virulence, and polymerase activity, indicating that the functional role of the single amino acid residue 431 in the PB2 protein was not strain specific.

In conclusion, our virological surveillance results demonstrated that SIVs of multiple genotypes and diverse virulence cocirculate in a specific region, which makes the epidemiology of swine influenza more complex and emphasizes the importance of continuous monitoring of influenza viruses in pigs. Also, we identified a novel virulence marker at residue 431 in PB2 of the H1N1 SIVs that significantly affects virulence in mice. Whether this amino acid substitution affects viral transmission requires further investigation.

## MATERIALS AND METHODS

### Ethics statement and facility.

The present study was carried out in strict accordance with the recommendations in the Guide for the Care and Use of Laboratory Animals of the Ministry of Science and Technology of the People’s Republic of China. All studies with live viruses were carried out in a biosecurity level 2 laboratory approved for such use by the Harbin Veterinary Research Institute (HVRI) of the Chinese Academy of Agricultural Sciences (CAAS). The protocol was approved by the Committee on the Ethics of Animal Experiments of the HVRI of the CAAS.

### Sample collection and virus isolation.

Between November 2014 and December 2016, a total of 5,750 nasal swabs were collected from pigs in slaughterhouses located in Liaoning Province, China. The samples were placed in a transport medium that consisted of phosphate-buffered saline (PBS) containing penicillin (2000 U/ml) and streptomycin (2,000 μg/ml). All of the individual samples were inoculated into 10-day-old embryonated chicken eggs for 72 h at 36°C. The allantoic fluid was collected and tested for hemagglutination activity with 0.5% chicken red blood cells. When the assay was positive, reverse transcription-PCR (RT-PCR) was then performed to further identify HA and NA subtypes, as described previously (11).

### Full-length sequencing and phylogenetic analyses.

The identified viruses were subjected to whole-genome sequencing and phylogenetic analyses. Viral RNA (vRNA) was extracted from virus-infected allantoic fluid using a QIAamp viral RNA minikit (Qiagen, Germantown, MD, USA). cDNAs were synthesized from vRNAs by RT with the Uni12 primer and were amplified by PCR with primers complementary to the conserved promoter and noncoding regions of each gene segment (the primer sequences are available upon request). The coding sequences for the entire genomes of the viruses were sequenced on an Applied Biosystems 3500xL genetic analyzer at the HVRI. The DNA sequences were compiled and edited using Lasergene 7.1 software (DNAStar Inc., Madison, WI, USA). For phylogenetic analysis, in addition to the full genomes of all the viruses we sequenced in this study, those of the 247 H1N1 viruses previously isolated in mainland China were also downloaded from GISAID. The phylogenetic tree of the isolates was generated by the distance-based neighbor-joining method using software MEGA7 (www.megasoftware.net). The reliability of the tree was assessed by bootstrap analysis with 1,000 replicates. Horizontal distances are proportional to genetic distance. Phylogenetic analysis of the eight gene segments was based on alignments of the open reading frames of the respective fragment.

### Viral infection in mouse model.

Ten isolates, including 9 H1N1 and 1 H1N2, were chosen to test their pathogenicity in mice. The virus stocks were prepared by propagating in 10-day-old specific-pathogen-free (SPF) embryonated chicken eggs, followed by determining the EID_50_ as described previously (15). A total of 88 6-week-old female BALB/c mice (Vital River Laboratories, Beijing, China) were randomly divided into 11 groups, of which 10 groups were i.n. inoculated with 10^5^ EID_50_ of each virus in a 50-μl volume. The remaining mice were i.n. inoculated with 50 μl of PBS as a mock control. All mice were weighed daily after infection. Three mice from each group were euthanized on 3 dpi to evaluate viral replication in the organs. At necropsy, brain, NT, lung, spleen, and kidney were collected and titrated in 10-day-old embryonated chicken eggs. The remaining mice were observed for clinical signs, their body weights were measured until 14 dpi, and mice that lost more than 25% of their initial body weight were euthanized on humane grounds. The serum samples were collected from mice at 14 dpi for seroconversion confirmation.

### Virus rescue.

Based on the pathogenesis of the isolates in mice, a pair of viruses possessing similar genetic characteristics but exhibiting different virulence in mice was chosen as model viruses to further investigate the factors involved in viral pathogenicity. First, RGSs for the two parent viruses were established by inserting the full-length cDNA derived from the indicated virus into the vRNA-mRNA bidirectional transcription vector pHW2000 (the plasmid was kindly provided by Richard Webby at St. Jude Children’s Hospital, Memphis, TN, USA) as described previously (59). Then, a series of reassortants were generated by a single-gene substitution and site-directed mutagenesis. Recombinant viruses were rescued by transfection in HEK293T cells and propagated in 10-day-old SPF embryonated chicken eggs, followed by whole-genome sequencing to ensure the absence of unwanted mutations. The sequences of primers for virus rescue are available upon request.

### Viral replication kinetics *in vitro*.

To determine the growth of the parental and recombinant viruses *in vitro*, MDCK cells and A549 cells were infected with the indicated viruses at a multiplicity of infection (MOI) of 0.001 and 0.1, respectively. After 1 h, MDCK cells were incubated at 37°C in Dulbecco’s modified Eagle’s medium (DMEM) containing 1 μg/ml tolylsulfonyl phenylalanyl chloromethyl ketone (TPCK)-treated trypsin and A549 in Ham’s F-12K medium containing 0.25 μg/ml of TPCK-treated trypsin. Supernatants were collected 12, 24, 36, 48, 60, and 72 h after infection and stored at –80°C. The viral titers were determined by endpoint titration in MDCK cells and expressed as mean log_10_ TCID_50_/ml ± standard deviation (SD).

### Viral replication capacity *in vivo*.

To evaluate the replication capacity of each virus *in vivo*, we euthanized three mice at 3 dpi with 10^6^ EID_50_ of each indicated virus, harvested the organs (including NT and lung), and titrated for virus infectivity in 10-day-old embryonated chicken eggs. Titers were calculated by the Reed-Muench method (60) and expressed as mean log_10_ EID_50_/ml ± SD. A value of 0.5 was assigned to virus titration-negative samples for statistical purposes.

### Determination of the MLD_50_.

The MLD_50_ values of the two parent wt viruses and all the rescued viruses were determined by inoculating i.n. groups of 5 BALB/c mice with 10-fold serial dilutions (10^1^ to 10^6^ EID_50_) of viruses in 50 μl of PBS. Body weight was measured daily for 14 dpi, and the mice that lost more than 25% of their original weight were euthanized for humane reasons. The MLD_50_ values were calculated by the Reed-Muench method (60) after a 14-day observation period and were expressed as log_10_ EID_50_.

### Polymerase activity analysis.

Polymerase activity was measured using a minigenome assay as previously described (61). In brief, subconfluent monolayers of HEK293T cells were transfected with a luciferase reporter plasmid (pPolI-Luc) together with the pRL-TK (Promega, Madison, WI, USA) and pcDNA3.1(+) plasmid constructs with various combinations of the polymerase genes PB2 (parental or mutant), PB1, PA, and NP using Lipofectamine LTX and Plus reagent (Thermo Fisher Scientific, Waltham, MA, USA) as recommended by the manufacturer. pRL-TK expresses *Renilla* luciferase and was used as an internal transfection control for the dual-luciferase assay. After 6 h of incubation at 37°C, the medium was replaced with DMEM containing 10% fetal bovine serum. Cell extracts were harvested 24 h posttransfection, and luciferase activity was assayed by using the Dual-Luciferase reporter assay system (Promega, Madison, WI, USA). Polymerase activity was calculated by standardization of the firefly luciferase activity to the *Renilla* luciferase activity. The polymerase activity of the wt-SY130 RNP was set as 100%. Experiments were performed in triplicate.

### Statistical analysis.

Data were analyzed using analysis of variance (ANOVA) in GraphPad Prism version 8.0 (GraphPad Software Inc., CA, USA). A probability (*P*) value of <0.05 was considered to be a statistically significant difference, while a *P* value of <0.01 was considered to be a highly significant difference. Two-way ANOVA followed by Tukey’s multiple-comparison test and ordinary one-way ANOVA were used.

### Data availability.

The eight gene segments of the isolates were sequenced and have been deposited in GenBank under accession numbers MN393701 to MN393876.
